# Efficient Charge Transfer Channels in Reduced Graphene Oxide/Mesoporous TiO_2_ Nanotube Heterojunction Assemblies toward Optimized Photocatalytic Hydrogen Evolution

**DOI:** 10.3390/nano12091474

**Published:** 2022-04-26

**Authors:** Zhenzi Li, Decai Yang, Hongqi Chu, Liping Guo, Tao Chen, Yifan Mu, Xiangyi He, Xueyan Zhong, Baoxia Huang, Shiyu Zhang, Yue Gao, Yuxiu Wei, Shijie Wang, Wei Zhou

**Affiliations:** Shandong Provincial Key Laboratory of Molecular Engineering, School of Chemistry and Chemical Engineering, Qilu University of Technology (Shandong Academy of Sciences), Jinan 250353, China; zzli@qlu.edu.cn (Z.L.); decaiyang@qlu.edu.cn (D.Y.); hongqichu@qlu.edu.cn (H.C.); lipingguo@qlu.edu.cn (L.G.); taochen@qlu.edu.cn (T.C.); yifanmou@qlu.edu.cn (Y.M.); xiangyihe@qlu.edu.cn (X.H.); xueyanzhong@qlu.edu.cn (X.Z.); baoxiahuang@qlu.edu.cn (B.H.); shiyuzhang@qlu.edu.cn (S.Z.); yuegao123@qlu.edu.cn (Y.G.); yuxiuwei@qlu.edu.cn (Y.W.)

**Keywords:** TiO_2_, photocatalysis, mesoporous structure, assembly, heterojunction

## Abstract

Interface engineering is usually considered to be an efficient strategy to promote the separation and migration of photoexcited electron-hole pairs and improve photocatalytic performance. Herein, reduced graphene oxide/mesoporous titanium dioxide nanotube heterojunction assemblies (rGO/TiO_2_) are fabricated via a facile hydrothermal method. The rGO is anchored on the surface of TiO_2_ nanosheet assembled nanotubes in a tightly manner due to the laminated effect, in which the formed heterojunction interface becomes efficient charge transfer channels to boost the photocatalytic performance. The resultant rGO/TiO_2_ heterojunction assemblies extend the photoresponse to the visible light region and exhibit an excellent photocatalytic hydrogen production rate of 932.9 μmol h^−1^ g^−1^ under simulated sunlight (AM 1.5G), which is much higher than that of pristine TiO_2_ nanotubes (768.4 μmol h^−1^ g^−1^). The enhancement can be ascribed to the formation of a heterojunction assembly, establishing effective charge transfer channels and favoring spatial charge separation, the introduced rGO acting as an electron acceptor and the two-dimensional mesoporous nanosheets structure supplying a large surface area and adequate surface active sites. This heterojunction assembly will have potential applications in energy fields.

## 1. Introduction

In recent years, the energy crisis and environmental pollution have become two major topics in the field of science and technology research [1,2]. The development and utilization of new technology has become the primary task of current research. Among various options, photocatalysis is favored for its low carbon footprint and use of renewable resources [3,4,5]. The most important thing is that it can directly use solar energy to generate new energy, such as hydrogen energy, and plays an important role in the degradation of pollutants and nitrogen fixation [6]. Therefore, it is considered to be an efficient strategy to solve the current increasingly serious environmental problems, and it has good application prospects.

In 1972, Honda and Fujishima [7] discovered that titanium dioxide (TiO_2_) could cause photocatalytic water-splitting. Since then, many scholars have begun to conduct a large number of experimental studies on TiO_2_ [8]. So far, TiO_2_ has obviously become the most widely studied photocatalyst due to its advantages of high stability and activity, super hydrophilicity, low cost and environmental friendliness in the field of photocatalysis [9,10]. However, from the perspective of the mechanism, it can be found that TiO_2_ has three fatal defects, which seriously limit its development. Firstly, TiO_2_ has a large band gap energy (3.2 eV), which means that the photogenerated electron-hole can only be activated by ultraviolet light with an energy higher than 3.2 eV (wavelength less than 387 nm), which only accounts for ~5% of sunlight. It was clear that visible light with a relatively long wavelength (almost 43% of sunlight) could not stimulate the photocatalytic activity of TiO_2_ [11,12]. Secondly, the excited photogenerated electrons and holes in TiO_2_ have a strong oxidability and reducibility, which leads to a rapid recombination and inefficient quantum efficiency. Studies have shown that most (about 90%) of the excited photogenerated electrons and holes of TiO_2_ have recombined before photocatalysis [13]. Thirdly, traditional TiO_2_ materials have a low specific surface area, which restricts the photocatalytic activity of TiO_2_ obviously [14,15]. 

In recent years, some methods have been adopted to improve the photocatalytic performance of TiO_2_. Firstly, the surface area of TiO_2_ could be improved by adjusting the morphology [16]. In addition, an excellent morphology can also improve the probability of charge transport by shortening the transmission path [17]. Secondly, TiO_2_ is modified by doping, metal deposition, and recombination to improve its activity. Doping can be used for improving the charge transfer efficiency and can be used for dragging down the bandgap [18]. Metal deposition can either form a Schottky barrier or an Ohmic contact, improving the efficiency of charge separation [19], and the introduction of metal can also broaden the absorption of light into the visible light region [20]. Recombination can improve charge separation efficiency through electron transfer [21]. Among various methods, heterojunction construction is a simple and practical method to effectively promote charge separation [22,23,24]. Graphene, as a representative of two-dimensional carbon materials, is composed of a single layer of carbon atoms with an sp^2^ hybrid orbital in the shape of a hexagonal honeycomb [25]. Graphene oxide (GO) is prepared by embedding O atoms into the c-scaffold of graphene to make sp^2^ and sp^3^ domains exist simultaneously in the structure, which promotes the expansion of multiple interactions. On this basis, reduced graphene oxide (rGO) can provide more interfacial polarization sites and absorption sites [26]. Thus, rGO has obvious application prospects in many fields including energy and environment, along with thermal and electrical properties [27,28,29]. In recent years, some reports had been reported on the application of rGO/TiO_2_ composite materials in the field of photocatalysis. Balsamo et al. successfully prepared a TiO_2_-rGO composite structure using the one-pot method by solar irradiation, which is simple and green. The degradation rate of 2,4-dichlorophenoxyacetic acid after irradiation for 3 h reached 97%. The enhanced absorption of the composite is due to the interaction between the TiO_2_ and rGO [30]. Zouzelka et al. optimized rGO/TiO_2_ composites by electrophoretic deposition. The presence of rGO can, to some extent, promote the photocatalytic degradation of 4-chlorophenol. Compared with TiO_2_, the photocatalytic degradation rate of the composite material is several times higher, and this enhanced performance is due to the charge transfer promoting the formation of hydroxyl radical [31]. Firstly, rGO could change the band gap width of TiO_2_ to expand its light response range. Secondly, it can be used as an electron transporter, which can effectively prevent photogenerated electron hole recombination and significantly prolong the lifetime of the electron-hole pair. In addition, a large specific surface area can provide more active sites for photocatalytic reactions [32]. Therefore, rGO/TiO_2_ composite materials maybe have good application prospects in the field of photocatalysis due to their high charge separation efficiency. Although these rGO/TiO_2_ composites indeed improved the photocatalytic performance obviously, they are still far from being practical applications. The question of how to further promote the charge separation of rGO/TiO_2_-based materials via interface engineering is still a great challenge.

Here, novel reduced graphene oxide/mesoporous titanium dioxide nanotube heterojunction assemblies (rGO/TiO_2_) are fabricated via a facile hydrothermal method by utilizing mesoporous TiO_2_ nanosheet-assembled nanotubes as the host. For one thing, the morphology of TiO_2_ was regulated into the nanotube structure assembled by the mesoporous nanosheet, which increased the specific surface area of the material and exposed more active sites. Besides, a tubular structure increased the multiple reflection of sunlight inside the tube and improved the utilization rate of light. For another thing, rGO, as an electron acceptor, was introduced to form rGO/TiO_2_ heterojunction assemblies, which contributed to improve the spatial charge separation efficiency and extend the photoresponse to the visible light region. The rGO/TiO_2_ heterojunction assemblies significantly improved the photocatalytic activity, and the photocatalytic hydrogen production rate was up to 932.9 umol h^−1^ g^−1^, 1.2 times higher than that of pristine TiO_2_. The novel rGO/TiO_2_ heterojunction assemblies may provide new insights for the fabrication of TiO_2_-based photocatalysts with a high performance.

## 2. Materials and Methods

### 2.1. Chemicals

Titanium oxysulfate (TiOSO_4_), potassium permanganate (KMnO_4_), sodium nitrate (NaNO_3_) and sulfuric acid (H_2_SO_4_) were purchased from Aladdin Chemical (Shanghai, China). Ethanol (EtOH), ether (C_2_H_5_OC_2_H_5_) and glycerol (C_3_H_5_(OH)_3_) were purchased from Tianjin Kermio Chemical (Tianjin, China). None of the chemicals were further purified.

### 2.2. Preparation of TiO_2_ Nanotube

The typical method for the synthesis of a TiO_2_ nanotube is based on the literature, and the specific steps are as follows [33]. 1 g TiOSO_4_ was added to 18 mL EtOH with intense stirring for 30 min, after which 9 mL C_3_H_5_(OH)_3_ and 9 mL C_2_H_5_OC_2_H_5_ were added drop by drop, stirred overnight, and then transferred to the Teflon reactor at 170 °C for 10 h. After the reaction, the obtained precursor was collected by repetitive centrifugation at 8000 rpm for 10 min and washed several times by deionized water and ethanol and dried at 60 °C for 12 h. After drying, the samples were transferred to a Muffle furnace and kept at 600 °C for 4 h. Finally, the TiO_2_ nanotube sample was obtained.

### 2.3. Preparation of rGO/TiO_2_

**Figure 1:**
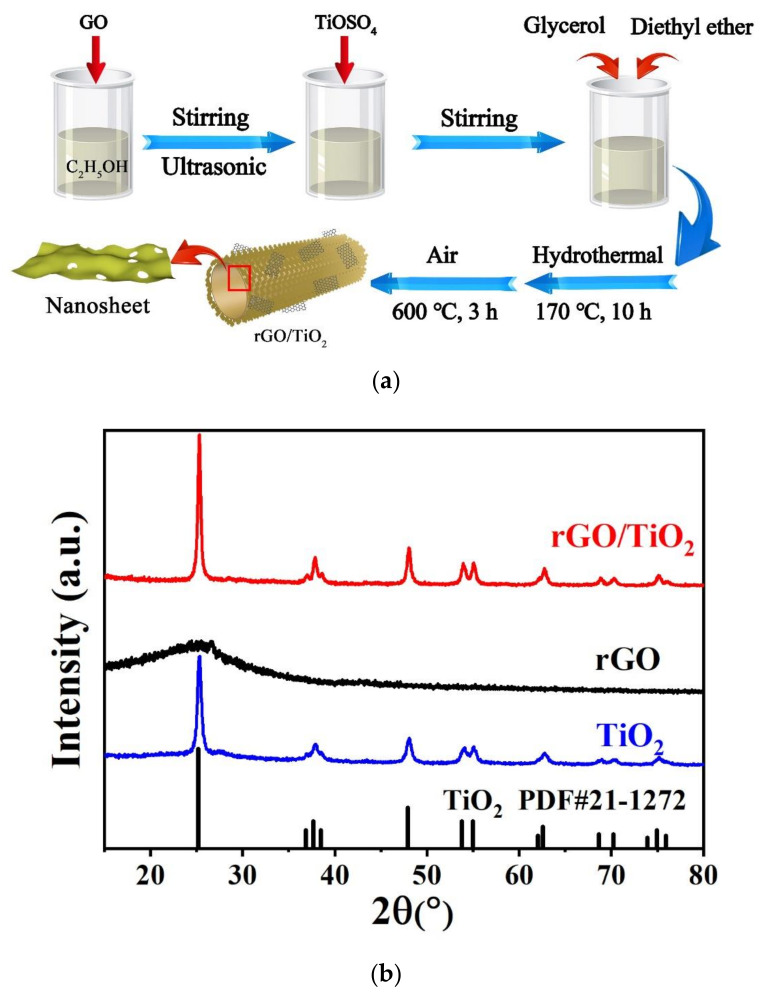

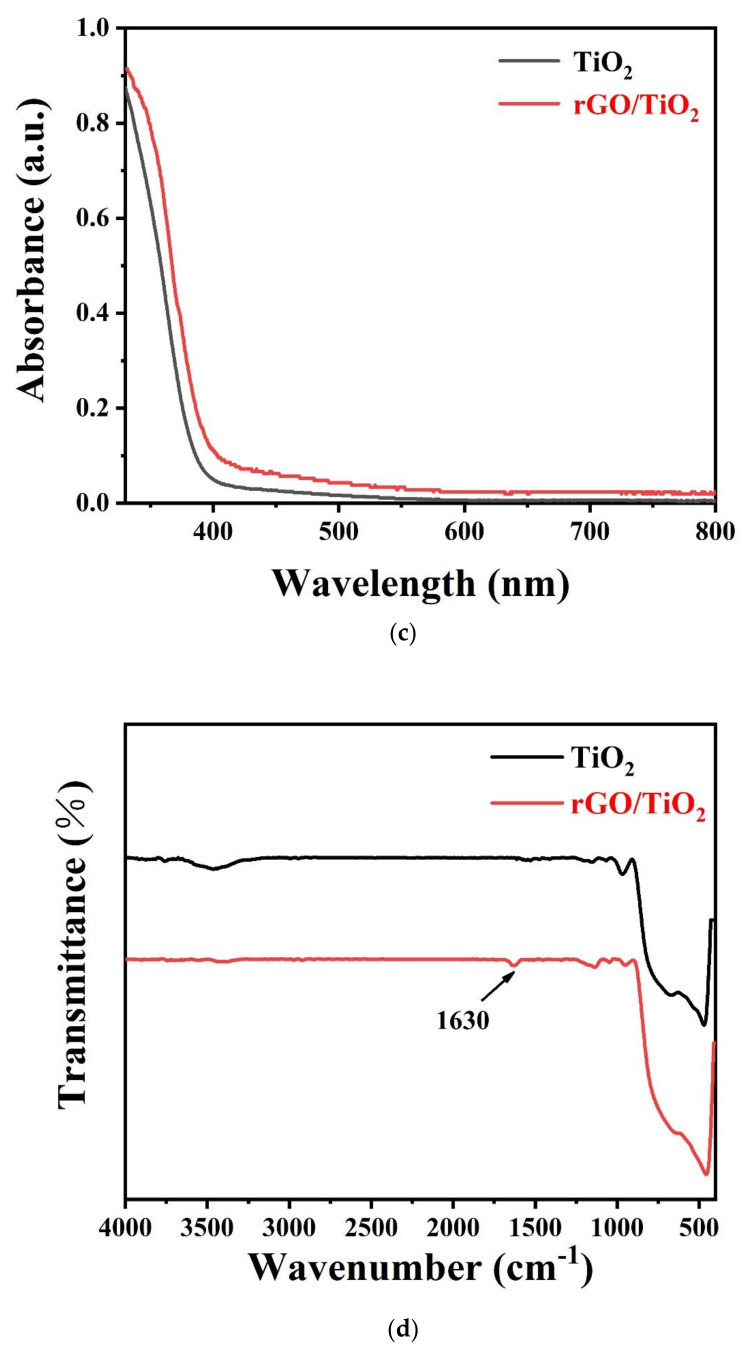
Schematic representation of the formation of (**a**) rGO/TiO2 heterojunction assembly pho-tocatalysts; (**b**) X-ray diffraction (XRD) patterns, (**c**) UV–visible diffuse reflectance spectra, and (**d**) FTIR of TiO_2_ and rGO/TiO_2_, respectively.

The formation process of rGO/TiO_2_ is shown in Figure 1a. rGO/TiO_2_ samples are synthesized on the basis of TiO_2_, and rGO is prepared by the hummer method using KMnO_4_, NaNO_3_ and H_2_SO_4_ as raw materials [34,35]. The prepared 5 mg rGO was added to 18 mL EtOH, stirred and ultrasonicated for 20 min, after which 1 g TiOSO_4_ was added. Then, 9 mL C_3_H_5_(OH)_3_ and 9 mL C_2_H_5_OC_2_H_5_ were added drop by drop, stirred overnight, and transferred to the Teflon-lined autoclave at 170 °C for 10 h. After the reaction, the resulting product was centrifuged and washed with deionized water and anhydrous ethanol 3 times and dried for later use. After drying, the samples were transferred to a Muffle furnace for calcination at 600 °C for 4 h. Finally, rGO/TiO_2_ samples were obtained. According to the feeding ratio, the content of rGO on TiO_2_ is 1 wt%.

## 3. Results and Discussion

### 3.1. Crystal Structure and Optical Absorption of rGO/TiO_2_

Figure 1b shows the X-ray diffraction (XRD) patterns of GO, TiO_2_ and GO/TiO_2_. The multiple diffraction peaks located at 25.3, 37.8, 48.0, 55.1, 62.8, 70.2 and 75.0° can be assigned to the (101), (004), (200), (211), (204), (116) and (220) planes of anatase TiO_2_ (JCPDS 21-1272), respectively [36]. This indicates the successful preparation of anatase TiO_2_. The characteristic peak positions of rGO (Figure 1b and Figure S1) are about 26° and 42.5°, which is consistent with the literature [37]. Figure 1b shows the XRD pattern of the rGO/TiO_2_ composite material, which is basically consistent with that of TiO_2_, indicating that the introduction of rGO does not destroy the crystal structure of TiO_2_. This also implies the stability of the material. However, the increased strength of the (101) crystal plane preliminarily indicates the successful introduction of rGO. The reason for this stems from the coincidence of the position of the TiO_2_ (101) crystal plane (25.3°) and rGO (26°), and no single peak of rGO can be observed. As shown in Figure 1c, UV-vis was applied to obtain the absorption property of the materials. TiO_2_ has a strong light absorption capacity when the wavelength is less than 400 nm, which is the inherent absorption property of crystal anatase TiO_2_. The absorption intensity of rGO/TiO_2_ was higher than that of TiO_2_, indicating the enhancement of the composites. The photoresponse region of the material extended the UV absorption to the visible light region, which was attributed to the modification of rGO. Figure 1d shows FTIR of TiO_2_ and rGO/TiO_2_, the principal vibration of TiO_2_ corresponding to the wide band in the range of 400−800 cm^−1^. The significant band at 670 cm^−1^ corresponds to the bending vibration mode of the Ti–O–Ti bond. For the rGO/TiO_2_ heterostructure, the wide absorption at low frequencies is similar to the spectrum of the TiO_2_ and can therefore be attributed to the vibration of the TiO_2_. In addition, the peak broadening of rGO/TiO_2_ relative to TiO_2_ below 1000 cm^−1^ may be due to the superposition of the peak existing in the Ti–O–C vibration (798 cm^−1^) on Ti–O–Ti. The emerging absorption band at around 1630 cm^−1^ can be associated with the bone vibration of the graphene sheet, indicating the successful loading of rGO [38]. The weak peak was due to the low amount of rGO on the TiO_2_ surface (about 1 wt%).

### 3.2. Morphology of rGO/TiO_2_ Assembly

**Figure 2:**
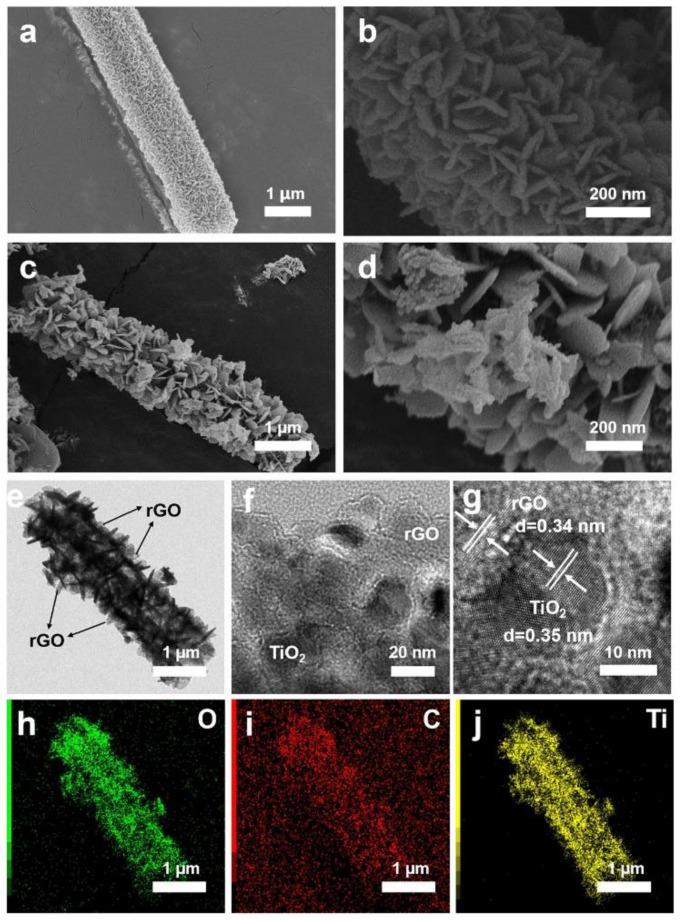
The SEM images of (**a**,**b**) TiO_2_ and (**c**,**d**) rGO/TiO_2_, and the (**e**) TEM, (**f**,**g**) HRTEM images and (**h**–**j**) elemental mappings of the rGO/TiO_2_ heterojunction assembly.

A scanning electron microscope (SEM, Hitachi, S-4800, Tokyo, Japan) and transmission electron microscope (TEM, JEOL, JEM-2100, Tokyo, Japan) were used to observe the microstructure of TiO_2_ and rGO/TiO_2_. It can be clearly seen that TiO_2_ shows a very uniform tubular structure, assembled by many sheets, with a diameter of about 700 nm (Figure 2a,b). Compared with TiO_2_, the surface of rGO/TiO_2_ becomes slightly rough, and the diameter of the tube increases slightly (Figure 2c,d). The sheet structure can increase the specific surface area and expose more active sites. The tubular structure formed by the self-assembly of multiple nanosheets can further increase the specific surface area of the material. Secondly, the tubular structure can reflect and utilize the sunlight efficiently, which improves the utilization rate of light and is conducive to improving the photocatalytic activity. In addition, the heterostructure formed by TiO_2_ and rGO is beneficial to the electron transfer between materials, which reduces the electron-hole recombination rate and improves the charge separation efficiency and photocatalytic activity. TEM images of rGO/TiO_2,_ as shown in Figure 2e, illustrate a nanosheet-assembled tubular structure, and the light-colored part on the surface is a layer of rGO, which is consistent with the results obtained by SEM. The rGO could be anchored on the surface of the nanosheets due to the laminated effect, thus forming efficient heterojunctions. The HRTEM images (Figure 2f,g) of rGO/TiO_2_ show that the edge of the sample is a layer of rGO and that the inner layer is TiO_2_. As shown in Figure 2g, clear lattice fringes can be seen with a lattice spacing of about 0.35 nm corresponding to the (101) crystal plane of anatase TiO_2_. Meanwhile, it can be clearly seen that the lattice spacing of rGO is 0.34 nm, which further indicates the successful preparation of the rGO/TiO_2_ heterojunction assembly material. Elemental mapping (Figure 2h–j) shows that O, C and Ti elements in rGO/TiO_2_ are evenly dispersed. The above information demonstrates the successful manufacturing of the rGO/TiO_2_ heterojunction assembly.

### 3.3. Photocatalytic Performance of rGO/TiO_2_

**Figure 3:**
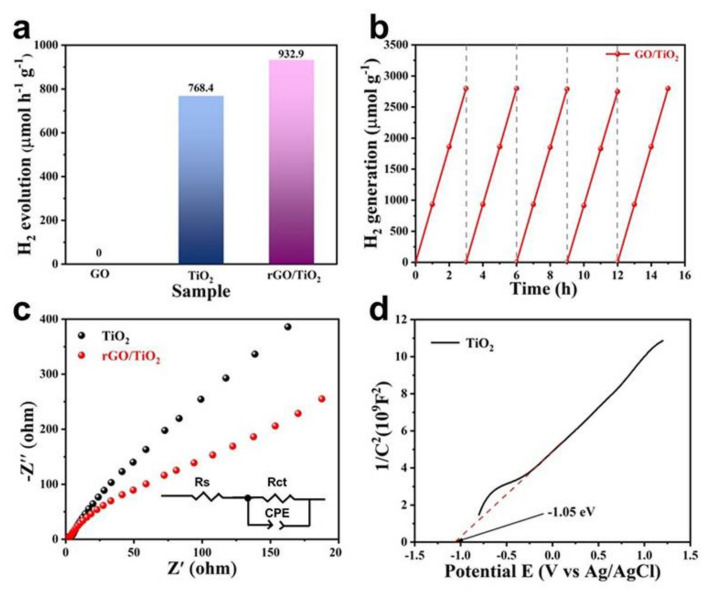
(**a**) Photocatalytic H_2_ evolution rate of rGO, TiO_2_ and rGO/TiO_2_, (**b**) recycling tests of rGO/TiO_2_, (**c**) the Nyquist plots of electrochemical impedance under AM 1.5G of TiO_2_ and rGO/TiO_2_, and (**d**) the Mott–Schottky plots of TiO_2_.

The photocatalytic performance of the catalysts for hydrogen production under favorable conditions of the catalyst Pt was conducted. As shown in Figure 3a, the hydrogen production performance of rGO is nearly 0, indicating that the presence of rGO does not contribute to hydrogen production, which is consistent with the reports in the literature. The hydrogen production quantity of rGO/TiO_2_ is 932.9 umol h^−1^ g^−1^, which is 1.2 times higher than that of original TiO_2_ (768.4 umol h^−1^ g^−1^). We can clearly see the excellent photocatalytic performance of rGO/TiO_2_, which is significantly better than in the previous literature (Table S1). This is mainly attributed to the formation of a heterojunction between TiO_2_ and rGO, which promotes the spatial charge separation and inhibits the recombination of electron-hole pairs. Secondly, the design of an rGO sheet supported on a TiO_2_ sheet is more conducive to external proton reduction. This 2D/2D design can also maximize the interface contact area, integrate the advantages of each 2D component, and facilitate the photocatalytic reaction. Finally, the nanosheet-assembled tubular structure provides a large specific surface area and sufficient surface active sites for the photocatalytic reaction. Under sunlight irradiation (AM 1.5G), the special microstructure can realize multiple reflections within the tubular structure, improving the utilization rate of sunlight. To investigate the stability of catalysts, cyclic tests were performed (Figure 3b). Five cycles of rGO/TiO_2_ were tested, and the hydrogen production rates were almost identical for each cycle. Besides, the amount of hydrogen production was not attenuated, indicating that rGO/TiO_2_ possessed a high stability, which was favorable for practical applications.

Figure 3c shows the electrochemical impedance spectra (EIS, Shanghai Chenhua Instrument Co., Ltd., CHI760E, Shanghai, China) of TiO_2_ and rGO/TiO_2_ under AM 1.5G light irradiation. It can be clearly seen that the arc radius of the EIS diagram of rGO/TiO_2_ is smaller than that of TiO_2_, indicating that rGO/TiO_2_ has a lower electrochemical impedance under light irradiation. This result clearly indicates that there is a positive synergistic effect between TiO_2_ and rGO on the enhancing electron migration, which has a more efficient charge carrier separation and faster electron transfer to reduce the electron-hole recombination rate and promote the photocatalytic hydrogen generation [39]. In order to analyze the semiconductor type and band structure of TiO_2_, the Mott–Schottky curve was tested for TiO_2_. As shown in Figure 3d, the slope of the curve is positive, which is typical for n-type semiconductors [40]. At the same time, the flat band potential of TiO_2_ (−1.05 eV) can be obtained from the slope of the curve, and the conduction band of TiO_2_ is −0.81 eV [41]. Combined with UV-vis, the valence band position is determined at 2.31 eV.

### 3.4. Photocatalytic Mechanism of rGO/TiO_2_

**Scheme 1:**
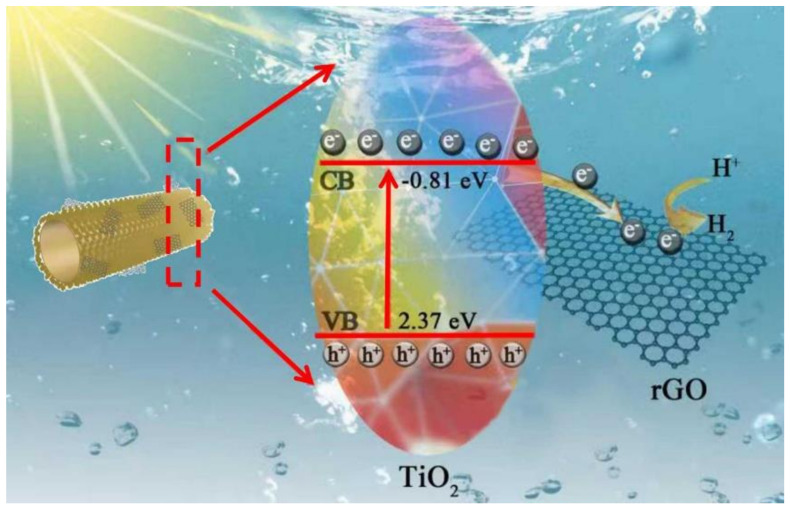
The mechanism diagram of photocatalytic hydrogen production of the rGO/TiO_2_ heterojunction assembly.

Based on the above, the mechanism diagram of photocatalytic hydrogen production of the rGO/TiO_2_ heterojunction was made, as shown in Scheme 1. When the rGO/TiO_2_ catalyst is irradiated by light, the electrons on TiO_2_ are excited to the conduction band (CB), leaving holes in the valence band (VB). Meanwhile, TiO_2_ will come into contact with rGO to form a built-in electric field. Since rGO is an electron acceptor, electrons will transfer from TiO_2_ CB to rGO. Additionally, the reduction reaction H^+^ converts to H_2_ on rGO. The improved photocatalytic performance is attributed to an electron derived from the rGO/TiO_2_ heterojunction, which reduces the recombination and improves the spatial charge separation efficiency. Such high photocatalytic properties are inseparable from the structure itself. The nanosheet-assembled hollow tubular structures provide a large specific surface area and adequate surface active sites for photocatalytic reactions. In addition, the nanotube structure can improve the utilization rate of light energy. The reason for this is that light can be reflected and used multiple times in the cavity. These factors all contribute to improving the photocatalytic hydrogen evolution of the rGO/TiO_2_ heterojunction assembly.

## 4. Conclusions

In summary, an rGO/TiO_2_ heterojunction assembly was prepared by a step hydrothermal method, and the H_2_ production rate was much faster than that of the original TiO_2_ nanotubes under AM 1.5G irradiation. This was mainly decided by the following two aspects. Firstly, the rGO heterostructure with TiO_2_ promoted the electron transfer, reduced the electron recombination rate, and improved the spatial charge efficiency. The design of rGO sheets supported by TiO_2_ sheets is more conducive to the reduction of external protons. Secondly, the advantage of the special structure was that the nanosheet-assembled hollow tube provided a large specific surface area. The combination of the two 2D structures maximizes the interface contact area and exposes enough reaction sites on the surface, which is favorable for photocatalytic hydrogen production. This novel strategy provides new ideas for the fabrication of other heterojunction photocatalysts with an efficient solar energy conversion.

## Data Availability

Data presented in this article are available at request from the corresponding author.

## Supplementary Materials

The following supporting information can be downloaded at: https://www.mdpi.com/article/10.3390/nano12091474/s1. Characterizations, Photocatalytic activity, Photoelectrochemical measurements [42,43,44,45,46,47]. Figure S1: XRD patterns of rGO, Table S1: Comparison with other TiO_2_ and rGO composite catalysts.
