# Supplementary material for: Efficient Charge Transfer Channels in Reduced Graphene Oxide/Mesoporous TiO2 Nanotube Heterojunction Assemblies toward Optimized Photocatalytic Hydrogen Evolution
Source: Nanomaterials (Basel). 2022 Apr 26;12(9):1474. doi: 10.3390/nano12091474 (PMC9103938; doi:10.3390/nano12091474)
Supplement: Supplementary file 1 [file nanomaterials-12-01474-s001.zip › nanomaterials-1675938-supplementary.pdf]

Supplementary Information

Efficient Charge Transfer Channels in Reduced Graphene Oxide/Mesoporous TiO_2 Nanotube Heterojunction Assemblies Toward Optimized Photocatalytic Hydrogen Evolution

Zhenzi Li †, Decai Yang †, Hongqi Chu, Liping Guo, Tao Chen, Yifan Mu, Xiangyi He, Xueyan Zhong, Baoxia Huang, Shiyu Zhang, Yue Gao, Yuxiu Wei, Shijie Wang * and Wei Zhou *

Shandong Provincial Key Laboratory of Molecular Engineering, School of Chemistry and Chemical Engineering, Qilu University of Technology (Shandong Academy of Sciences), Jinan 250353, China; zzli@qlu.edu.cn (Z.L.); decaiyang@qlu.edu.cn (D.Y.)hongqichu@qlu.edu.cn (H.C.); lipingguo@qlu.edu.cn (L.G.); taochen@qlu.edu.cn (T.C.); yifanmou@qlu.edu.cn (Y.M.); xiangyihe@qlu.edu.cn (X.H.); xueyanzhong@qlu.edu.cn (X.Z.); baoxiahuang@qlu.edu.cn (B.H.); shiyuzhang@qlu.edu.cn (S.Z.); yuegao123@qlu.edu.cn (Y.G.); yuxiuwei@qlu.edu.cn (Y.W.)

* Correspondence: wsj0924@qlu.edu.cn (S.W.); wzhou@qlu.edu.cn (W.Z.)

† These authors contributed equally to this work.

Characterizations

X-ray diffraction (XRD) patterns were obtained by a Bruker D8 Advance diffractometer by using Cu Kα radiation (λ = 1.5406 Å, 40 kV, 40 mA). The step size and scan range were 0.02 ° and 5-80 °, respectively. The UV-visible absorption spectrum was measured using an UV-visible spectrophotometer (Shimadzu UV-2550). The surface morphology was measured by scanning electron microscopy (SEM) (Hitachi S-4800) and transmission electron microscopy (TEM) (JEOLJEM-2100).

Photocatalytic activity

The photocatalytic hydrogen production experiment was carried out in an online photocatalytic hydrogen production reaction system (AuLight, CEL-SPH$_2$N, Beijing Perfect Light). The temperature of the pipeline was set at 5 °C and the temperature of the reactor was set at 25 °C (to ensure the reaction activity of the catalyst). Before the reaction, 50 mg catalyst was added to the reactor. Then, 90 mL deionized water and 10 mL methanol (sacrificial agent) were selected as the reaction system, and a platinum source with a feed ratio of 3% ($H_2PtCl_6{\cdot}6H_2O$) was selected as the cocatalyst. Finally, the reactor was mixed evenly with a magnetic stirrer and loaded into a gas circulation reactor. Before the reaction, remove oxygen and carbon dioxide from the mixture with a vacuum pump. Xenon lamp with a power density of 100 mW cm^{-2} and a filter of AM 1.5G was used for optical deposition. After 30 min, the experiment was officially started and analyzed by online gas

chromatograph (SP7800, TCD, 5 Å molecular sieve, Ar carrier, Beijing Keruida Ltd., China). The same closed cycle system was evaluated the recycling capacity of the catalysts every 3 h. At the end of each cycle, 1 mL methanol was added to the reactor to ensure that the cocatalyst was sufficient, and subsequent cycles were started.

Photoelectrochemical measurements

Electrochemical workstation was used to analyze the photoelectrochemical properties of the samples. The instrument was CHI760E series double potentiostat from Shanghai Chenhua. Under the lighting condition, Xenon lamp (300 W, Beijing perfect Light) with AM 1.5G filter was used. The electrochemical workstation adopts a three-electrode system, with photoanode as catalyst, platinum electrode as cathode, Ag/AgCl electrode as reference electrode, and 0.5 M Na_2SO_4 solution was used as electrolyte. The photoanode was prepared as follows: 20 mg catalyst was dispersed into 30 mL ethanol solution, and then uniformly dispersed to the front of the FTO glass with a spray gun. The coated FTO glass was calcined at 300 °C for 2 h under N_2 atmosphere to prevent the catalyst from falling off during the test.

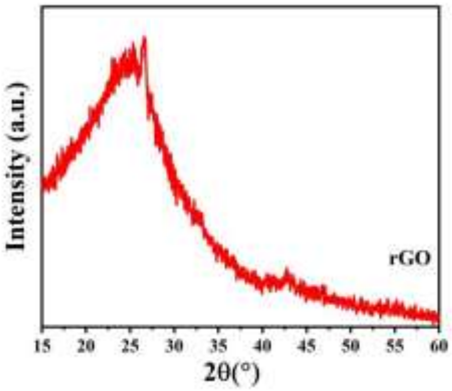

Figure S1. XRD patterns of rGO.

Table S1. Comparison with other TiO_2 and rGO composite catalysts

**Photocatalyst**	**Morphology of TiO_2**	**Construction**	**H_2 production rate**	**Refs.**
TiO_2-rGO	Sheet	TiO_2 on the rGO	About 600 umol h^{-1} g^{-1}	[42]
rGO-TiO_2	Out-of-shape	rGO on the TiO_2	About 120 umol h^{-1} g^{-1}	[43]
TiO_2-rGO	Sheet	TiO_2 on the rGO	About 450 umol h^{-1} g^{-1}	[44]
rGO-TiO_2	Sheet	rGO on the TiO_2	About 32 umol h^{-1} g^{-1}	[45]
V-TiO_2/RGO	Nanorod	TiO_2 on the rGO	About 530 umol h^{-1} g^{-1}	[46]
TiO_2@rGO	Sheet	TiO_2 on the rGO	About 0 umol h^{-1} g^{-1}	[47]
rGO/TiO_2	A hollow tube composed of sheets	rGO on the TiO_2	932.9 umol h^{-1} g^{-1}	This work

References

42. Gupta, B.; Melvin, A.; Matthews, T.; Dhara, S.; Dash, S.; Tyagi, A. Facile gamma radiolytic methodology for TiO_2-rGO synthesis: Effect on photo-catalytic H_2 evolution. *Int. J. Hydrogen Energ.* **2015**, *40*, 5815–5823.

43. Bharad, P.; Sivaranjani, K.; Gopinath, C. A rational approach towards enhancing solar water splitting: A case study of Au-RGO/N-RGO-TiO_2. *Nanoscale* **2015**, *7*, 11206–11215.

44 Ma, J; Dai, J.; Duan, Y.; Zhang, J.; Qiang, L.; Xue, J. Fabrication of PANI-TiO_2/rGO hybrid composites for enhanced photocatalysis of pollutant removal and hydrogen production. *Renew. Energ.* **2020**, *156*, 1008–1018.

45. Tudu, B.; Nalajala, N.; Reddy, K.; Saikia, P.; Gopinath, C. Electronic integration and thin film aspects of Au−Pd/rGO/TiO_2 for improved solar hydrogen generation. *ACS Appl. Mater. Inter.* **2019**, *11*, 32869−32878.

46. Agegnehu, A.; Pan, C.; Tsai, M.; Rick, J.; Su, W.; Lee, J.; Hwang, B. Visible light responsive noble metal-free nanocomposite of V-doped TiO_2 nanorod with highly reduced graphene oxide for enhanced solar H_2 production. *Int. J. Hydrogen Energ.* **2016**, *41*, 6752–6762.

47. Wei, X.; Cao, J.; Fang, F. A novel multifunctional Ag and Sr^{2+} co-doped TiO_2@rGO ternary nanocomposite with enhanced p-nitrophenol degradation, and bactericidal and hydrogen evolution activity. *RSC Adv.* **2018**, *8*, 31822–31829.
